# AI-Model for Identifying Pathologic Myopia Based on Deep Learning Algorithms of Myopic Maculopathy Classification and “Plus” Lesion Detection in Fundus Images

**DOI:** 10.3389/fcell.2021.719262

**Published:** 2021-10-15

**Authors:** Li Lu, Peifang Ren, Xuyuan Tang, Ming Yang, Minjie Yuan, Wangshu Yu, Jiani Huang, Enliang Zhou, Lixian Lu, Qin He, Miaomiao Zhu, Genjie Ke, Wei Han

**Affiliations:** ^1^Department of Ophthalmology, Eye Center of the Second Affiliated Hospital, School of Medicine, Zhejiang University, Hangzhou, China; ^2^Department of Ophthalmology, The First Affiliated Hospital, School of Medicine, Zhejiang University, Hangzhou, China; ^3^Department of Ophthalmology, The First Affiliated Hospital of University of Science and Technology of China, Hefei, China; ^4^College of Computer Science and Technology, Zhejiang University, Hangzhou, China

**Keywords:** artificial intelligence, deep learning, pathologic myopia, myopic maculopathy, “Plus” lesion, fundus image

## Abstract

**Background:** Pathologic myopia (PM) associated with myopic maculopathy (MM) and “Plus” lesions is a major cause of irreversible visual impairment worldwide. Therefore, we aimed to develop a series of deep learning algorithms and artificial intelligence (AI)–models for automatic PM identification, MM classification, and “Plus” lesion detection based on retinal fundus images.

**Materials and Methods:** Consecutive 37,659 retinal fundus images from 32,419 patients were collected. After excluding 5,649 ungradable images, a total dataset of 32,010 color retinal fundus images was manually graded for training and cross-validation according to the META-PM classification. We also retrospectively recruited 1,000 images from 732 patients from the three other hospitals in Zhejiang Province, serving as the external validation dataset. The area under the receiver operating characteristic curve (AUC), sensitivity, specificity, accuracy, and quadratic-weighted kappa score were calculated to evaluate the classification algorithms. The precision, recall, and F1-score were calculated to evaluate the object detection algorithms. The performance of all the algorithms was compared with the experts’ performance. To better understand the algorithms and clarify the direction of optimization, misclassification and visualization heatmap analyses were performed.

**Results:** In five-fold cross-validation, algorithm I achieved robust performance, with accuracy = 97.36% (95% CI: 0.9697, 0.9775), AUC = 0.995 (95% CI: 0.9933, 0.9967), sensitivity = 93.92% (95% CI: 0.9333, 0.9451), and specificity = 98.19% (95% CI: 0.9787, 0.9852). The macro-AUC, accuracy, and quadratic-weighted kappa were 0.979, 96.74% (95% CI: 0.963, 0.9718), and 0.988 (95% CI: 0.986, 0.990) for algorithm II. Algorithm III achieved an accuracy of 0.9703 to 0.9941 for classifying the “Plus” lesions and an F1-score of 0.6855 to 0.8890 for detecting and localizing lesions. The performance metrics in external validation dataset were comparable to those of the experts and were slightly inferior to those of cross-validation.

**Conclusion:** Our algorithms and AI-models were confirmed to achieve robust performance in real-world conditions. The application of our algorithms and AI-models has promise for facilitating clinical diagnosis and healthcare screening for PM on a large scale.

## Introduction

It is now widely believed that myopia is epidemic across the world, especially in developed countries of East and Southeast Asia (Dolgin, 2015). Myopia also has a significant impact on public health and socioeconomic wellbeing (Smith, 2009; Zheng et al., 2013; Holden et al., 2016). Pathologic myopia (PM), a severe form of myopia defined as high myopia combined with a series of characteristic maculopathy lesions, involves a greater risk of adverse ocular tissue changes leading to associated sight-threatening complications (Wong et al., 2014; Cho et al., 2016). For this reason, PM is a major cause of severe irreversible vision loss and blindness in East Asian countries (Morgan et al., 2017; Ohno-Matsui et al., 2019).

Due to the irreversible pathologic alterations in the shape and structure of the myopic globe, effective therapies for PM are still lacking, and the prognosis of PM complications is often poor. Moreover, as the disease process progresses slowly (Hayashi et al., 2010), PM patients often ignore their ocular symptoms and attribute these changes to their unsuitable glasses. Therefore, a better strategy for PM may be regular screening in myopic populations to identify and stop the aggravation of PM at an early stage. The precise diagnosis and evaluation of PM requires ophthalmic work-up and is aided by a series of imaging examinations, including fundus imaging, optical coherence tomography (OCT), and three-dimensional magnetic resonance imaging (3D-MRI) (Faghihi et al., 2010; Moriyama et al., 2011) which can hardly be included in screening programs. A recent meta-analysis of a pathologic myopia system (META-PM) provided a new simplified systematic classification for myopic maculopathy (MM) and defined PM based on fundus photography, which offers us a practical screening criterion (Ohno-Matsui et al., 2015). According to this classification standard, eyes with MM, which is equal to or more serious than diffuse choroidal atrophy, or with at least one “Plus” lesion, can be defined as having PM (Ohno-Matsui, 2017). However, even with this criterion, PM screening still depends on careful examination of the whole retina by retinal specialists through a magnified slit lamp noncontact lens or fundus images (Baird et al., 2020), challenging the ophthalmic medical resources in terms of clinical data analysis, especially retinal fundus image reading. It is difficult to imagine that such a large-scale screening task could be carried out by humans alone.

Fortunately, with the rapid development of artificial intelligence (AI) technologies, a sophisticated subclass of machine learning known as deep learning plays important roles in automated clinical data processing and hence makes labor-intensive work feasible (Hamet and Tremblay, 2017). The AI-model, with a deep artificial neural network as its core, has shown great efficiency and excellent performance comparable to those of board-certified specialists with respect to massive medical analysis (Esteva et al., 2017; Zhao et al., 2018; Liao et al., 2020). AI-model related diagnosis software has been successfully applied to screening tasks of diabetic retinopathy and glaucoma (Bhaskaranand et al., 2019; Li et al., 2020).

This study aimed to design and train a series of deep learning algorithms and AI-models based on the META-PM classification system using a large dataset of color retinal fundus images obtained from the ophthalmic clinics of hospitals and annotated by expert teams. We hope our models could (1) identify PM, (2) classify the category of MM, and (3) detect and localize the “Plus” lesions automatically. These works would facilitate the PM identification for either clinical management in hospital or healthcare service in community.

## Materials and Methods

### Data Collection

In this study, the use of images was approved by the Ethics Committee of First Affiliated Hospital, School of Medicine, Zhejiang University. As the study was a retrospective review and analysis of fully anonymized retinal fundus images, the medical ethics committee declared it exempt from informed consent.

Altogether, 37,659 original color retinal fundus images of 32,419 myopia patients were obtained from the eye centers of the First Affiliated Hospital of School of Medicine, Zhejiang University; the First Affiliated Hospital of University of Science and Technology of China; and the First Affiliated Hospital of Soochow University between July 2016 and January 2020, and analysis began February 2020. Three different desktop nonmydriatic retinal cameras (Canon, NIDEK, and Topcon) were used. Similar imaging protocols were applied for all three systems. All retinal fundus images were maculalutea-centered 45° color fundus photographs. Pupil dilation was decided by the examiners depending on the patient’s ocular condition. All patient data displayed with the images were pseudonymized before study inclusion.

Subsequently, the ungradable images were excluded. The criteria applied to determine a gradable image are listed as follows:

(a) Image field definition: primary field must include the entire optic nerve head and macula.
(b) Images should have perfect exposure because dark and washed-out areas interfere with detailed grading.
(c) The focus should be good for grading of small retinal lesions.
(d) Fewer artifacts: Avoid dust spots, arc defects, and eyelash images.
(e) There should be no other errors in the fundus photograph, such as the absence of objects in the picture.

According to this criteria, 5,649 ungradable images were excluded. A total dataset of 32,010 color retinal fundus images was established and further annotated by ophthalmologists.

### Definitions, Annotation, and the Reference Standard

According to the META-PM study classification, MM was classified into five categories: no myopic retinal degenerative lesion (Category 0), tessellated fundus (Category 1), diffuse chorioretinal atrophy (Category 2), patchy chorioretinal atrophy (Category 3), and macular atrophy (Category 4). Additionally, lacquer cracks (LCs), myopic choroidal neovascularization (CNV), and Fuchs’ spot were defined as “Plus” lesions (Ohno-Matsui et al., 2015). Thus, in the present study, fundus image with MM ≥ Category 2 or with at least one of the “Plus” lesions were considered as a PM image, while the remaining images were defined as non-PM images including the MM of Category 0 or Category 1 without “Plus” lesions. All the images of PM or C1-C4 MM were from high myopia patients whose spherical equivalence is worse than −6.0 D. The relevant demographic data were shown in Table 1. It is worth mentioning that Category 0 in this study included normal fundus and other fundus diseases.

**TABLE 1:** Summary of the total dataset and external validation dataset.

	Number of images	Number of	Number of	Mean age	Sex	Spherical Equivalent
	with labels	participants	ROI with labels	(years)	(% female)	(diopters)
**Total dataset**						
None PM	26,131	24,708	NA	50.39 ± 14.27 (24 to 81)	56.3	−2.07 ± 3.79 (−13 to −0.5)
Pathologic myopia	5,879	4,205	NA	52.39 ± 15.15 (23 to 82)	66.0	−13.72 ± 4.54 (−23.0 to −6)
Category 0	20,919	20,884	NA	50.52 ± 14.47 (26 to 75)	54.5	−0.53 ± 0.33 (−6.5 to −0.5)
Category 1	5,345	3,902	NA	49.59 ± 12.99 (24 to 81)	65.8	−10.52 ± 2.98 (−17.5 to −6)
Category 2	4,044	2,943	NA	50.10 ± 14.90 (23 to 75)	64.5	−12.88 ± 4.02 (−19.25 to −7.25)
Category 3	1,154	871	NA	56.90 ± 14.63 (29 to 80)	71.1	−15.77 ± 4.89 (−22.5 to −7.75)
Category 4	548	313	NA	63.22 ± 12.49 (33 to 82)	68.7	−16.21 ± 5.38 (−23.0 to −8.25)
CNV	734	442	857	55.28 ± 15.42 (26 to 82)	60.6	−15.02 ± 3.98 (−22 to -7)
Fuchs	746	411	3,020	58.24 ± 13.40 (24 to 80)	58.2	−16.21 ± 4.23 (−23 to −7.75)
LC	99	79	207	43.11 ± 12.76 (25 to 73)	56.3	−15.47 ± 3.09 (−21.75 to −6.75)
**External validation dataset**						
None PM	434	381	NA	51.28 ± 9.61 (16 to 69)	55.1	−4.92 ± 5.16 (−16 to −0.5)
Pathologic myopia	566	351	NA	54.71 ± 13.60 (17 to 83)	61.3	−15.41 ± 6.05 (−23.5 to −6)
Category 0	229	217	NA	50.85 ± 10.82 (16 to 69)	49.3	−0.67 ± 0.72 (−6.75 to −0.5)
Category 1	222	178	NA	50.21 ± 9.41 (18 to 67)	59.6	−10.81 ± 2.68 (−17.75 to −6)
Category 2	220	149	NA	51.60 ± 10.48 (17 to 75)	61.7	−14.02 ± 5.54 (−21 to −7.25)
Category 3	196	115	NA	55.93 ± 13.06 (26 to 80)	66.1	−16.35 ± 6.14 (−23.5 to −6.75)
Category 4	133	73	NA	63.71 ± 13.59 (35 to 83)	60.3	−17.04 ± 6.58 (−23.5 to −6.5)
CNV	67	41	97	51.27 ± 13.88 (27 to 80)	54.5	−16.15 ± 5.57 (−23 to −6.5)
Fuchs	205	130	878	59.24 ± 13.42 (27 to 81)	58.6	−16.89 ± 5.81 (−23.25 to −7)
LC	9	6	24	39.50 ± 11.26 (26 to 53)	50.0	−15.96 ± 3.78 (−22.25 to −7.5)

After learning the definition and testing the intra- and inter-rater reliability, a total of 20 ophthalmologists from three ophthalmic centers, who achieved a kappa value ≥0.81 (almost perfect), participated in manual grading and annotation and served as graders (Landis and Koch, 1977). Fifteen of them were general ophthalmologists with more than 5 years of experience, and five of them were senior retinal specialists with over 10 years of experience. They were randomly grouped into five teams, with each team having one senior specialist. The reference standard was determined based on the following protocol. Graders on the same team evaluated the same set of images. Each grader was blinded to the grades given by the others, and they made independent decisions on the fundus images. The results recognized unanimously by the three graders of the same team were taken as the reference standard. The results that differed among the general ophthalmologists in the same team were arbitrated by the retinal specialist for the final annotation decision. For the detailed workflow of data processing, all available fundus images were involved (*n* = 37,659) at the beginning stage, and the ungradable images were then identified and excluded by the grader teams. Next, in the gradable images group (*n* = 32,010), image-level binary classification label was given by grader teams to describe whether the eye had PM, which was used to develop algorithm I. Simultaneously, all the gradable images would obtain a category label according to its MM category, which was used to develop algorithm II. These two kinds of labels were based on the criteria of META-PM classification, with PM or C1–C4 MM images confirmed by the refractive error data (spherical equivalence worse than −6.0 D). Lastly, in the PM image group, graders localized the “Plus” lesions within the image if they existed by drawing rectangular bounding boxes, which was used to develop algorithm III. Meanwhile, the image was labeled as having the corresponding “Plus” lesions.

### Image Preprocessing and Augmentation

All the raw fundus images were preprocessed by cropping and resizing to a resolution of 512 × 512 pixels to meet the requirement of the input image format. Grayscale transformations, geometric variation, and image enhancement were applied to eliminate irrelevant information and recover useful or true information in the images.

### Development of the Deep Learning Algorithms and AI-Models

Our training platform was implemented with the PyTorch framework, and all of the deep learning algorithms were trained in parallel on four NVIDIA 2080 Ti graphics processing units (Paszke et al., 2019). In this study, three deep learning algorithms were trained after annotation: (I) for the binary classification of non-pathologic myopia/pathologic myopia (NPM/PM), (II) for the five-class classification of MM categories, and (III) for “Plus” lesion detection and localization.

**FIGURE 1:**
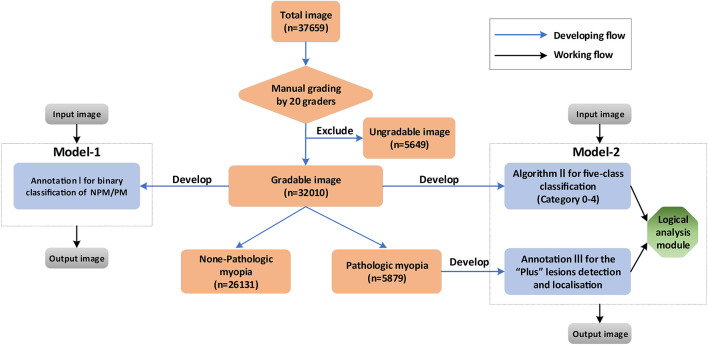
The diagram showing the detailed developing and working flow of our algorithms and artificial intelligence (AI)–models.

Based on the three algorithms, two AI-models, namely Model I and II, were developed. Model-1 was a one-step model, only containing algorithm I, to directly identify the NPM and PM. Model-2 was a two-step model, consisting of algorithm II, algorithm III, and a logical analysis module. The core of the logical analysis module was the META-PM classification. Step 1 was to obtain the output of the image by algorithm II and algorithm III, while step 2 was to use the logical analysis module to analyze the result of step 1 and then determine whether the image was of PM. The performance of two models was then compared in order to obtain the optimized model for PM identification. The detailed workflow is shown in Figure 1.

A five-fold cross-validation approach was employed to train and test the algorithms (Yang et al., 2019). The total dataset was randomly subgrouped into five equally sized folds at the image level, and each image was only allowed to exist in one-fold. Effort was made to ensure that the rate of classification outcome was basically consistent from fold to fold (Herzig et al., 2020). The development process included two steps: first, we randomly selected four-folds for algorithm training and hyperparameter optimization and the remaining fold for testing. Then, this process was repeated five times to confirm that each fold was set as the testing set (Keenan et al., 2019).

### Architecture of Deep Learning Algorithms

Algorithm I and algorithm II in this study were based on a state-of-the-art convolutional neural network (CNN) architecture, namely, ResNet18, while algorithm III for “Plus” lesion localization was constructed using a feature pyramid network (FPN)–based faster region-based convolutional neural network (Faster R-CNN). These architectures were all pretrained on the ImageNet dataset. The details of the relevant CNN architectures are shown in Supplementary Figure 1.

### Retrospective External Validation and Expert-Machine Comparison

To further evaluate our algorithms, we also retrospectively recruited 1,000 images from 732 patients from the three other hospitals in Zhejiang Province, serving as the external validation dataset (Table 1). Two different types of desktop nonmydriatic retinal cameras (Canon and ZEISS) were used to capture fundus images, and these were different from the cameras used to acquire training data. The annotation protocol for this dataset was the same as that for the total dataset. The images in the external validation dataset were simultaneously evaluated by the algorithms and two experts (one general ophthalmologist and one retinal specialist) who were not the participants in the aforementioned grading teams. The comparison results between the algorithms and experts were used to further quantify the performance of the algorithms.

### Misclassification and Visualization Heatmap Analysis of Classification Algorithms

In the external validation dataset, the images misclassified by algorithms I and II were further analyzed by a senior retinal specialist. To provide detailed guidance for clinical analysis, a convolutional visualization layer was implanted at the end of algorithm II. Then, this layer generated a visualization heatmap highlighting the strongly predictive regions on retinal fundus images (Gargeya and Leng, 2017). The consistency analysis between the hot regions and the actual lesions was evaluated by a senior retinal specialist.

### Statistics

According to the reference standard, all five-fold cross-validation results of the algorithms were recorded, and the average metrics were calculated. The performance of algorithm I was evaluated using the indices of sensitivity, specificity, accuracy, and area under the receiver operating characteristic curve (AUC). For the five-class classification of MM categories, the area under the macroaverage of ROC curve (macro-AUC) for each class in a one-vs.-all manner, the kappa score and the accuracy were calculated to evaluate algorithm II. Algorithm III was evaluated in two dimensions: (1) image classification and (2) region of interest (ROI) detection and lesion localization. Two groups of performance metrics were calculated. The former consisted of the accuracy, sensitivity, and specificity of binary classifications of the image with “Plus” lesions, while the latter included precision, recall, and F1-score. Model-1 and model-2 were compared with respect to the indices of sensitivity, specificity, precision, and accuracy. In the external validation dataset, the same indices were also calculated and compared with the experts of different expertise levels. All of the statistical tests in our study were two-sided, and a *P*-value less than 0.05 was considered significant. Additionally, the Clopper–Pearson method was used to calculate the 95% CIs. Statistical data analysis was implemented using IBM SPSS statistics for Windows version 26.0 (SPSS Inc., Chicago, IL, United States) and Python 3.7.3.

## Results

A total dataset of 32,010 color retinal fundus images from 28,913 patients was built and used for algorithm training and validation. Among the total dataset, approximately 13% of the graded images with inconsistent diagnoses were submitted to retinal specialists for final grading. The characteristics of the total dataset are summarized in Table 1.

### Performance of the Five-Fold Cross-Validation

**FIGURE 2:**
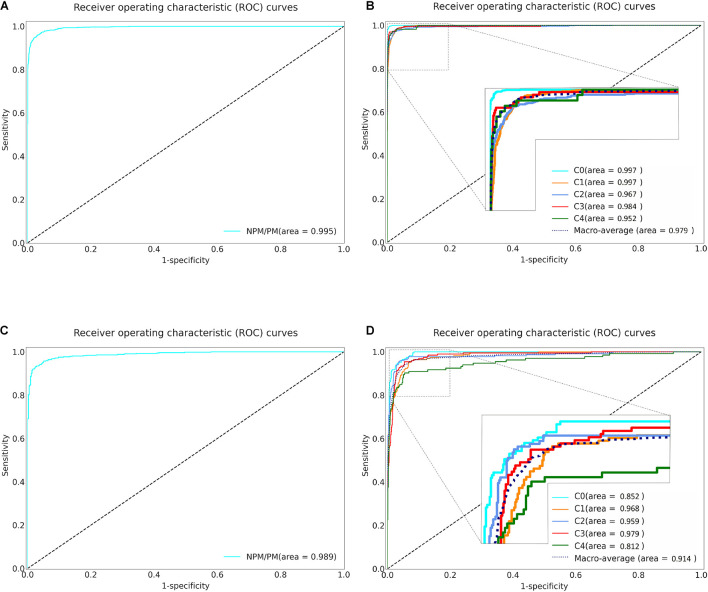
Receiver operating characteristic (ROC) curves of Algorithm I and Algorithm II in five-fold cross-validation and external validation. **(A)** The ROC curve of the algorithm I for identifying pathologic myopia in five-fold cross-validation. **(B)** The ROC curve of the algorithm II for classifying the category of MM in five-fold cross-validation. **(C)** The ROC curve of the algorithm I for identifying pathologic myopia in external validation. **(D)** The ROC curve of the algorithm II for classifying the category of MM in external validation. NPM: non-pathologic myopia. PM: pathologic myopia. area: area under the receiver operating characteristic curve. C: Category.

The five-fold cross-validation was used to evaluate the three algorithms. Specifically, algorithm I achieved an AUC of 0.995 (95% CI: 0.993–0.996), accuracy of 0.973 (95% CI: 0.969–0.977), specificity of 0.981 (95% CI: 0.978–0.985) and sensitivity of 0.939 (95% CI: 0.933–0.945) (Table 2 and Figure 2A). Algorithm II achieved a macro-AUC value of 0.979 (95% CI: 0.972–0.985), accuracy of 0.967 (95% CI: 0.963–0.971), and quadratic-weighted kappa of 0.988 (95% CI: 0.986–0.990) for differentiating the five MM categories (Table 2 and Figure 2B). From C0 to C4 MM, the specific accuracy of algorithm II is 97.7, 97.8, 91.3, 96.1, and 90.0% respectively. The confusion matrices of algorithm I and algorithm II are shown in Supplementary Figures 2A,B. Algorithm III achieved an accuracy of 0.970 to 0.994 for identifying the “Plus” lesions and an F1-score of 0.685 to 0.889 for detecting and localizing lesions. The typical output images of algorithm III are shown in Supplementary Figure 3. The more detailed results are listed in Table 2. The accuracy of model-1 and model-2 was 0.973 (95% CI: 0.969–0.977) and 0.984 (95% CI: 0.981–0.987), respectively. The two-step model-2 showed better performance in identifying PM.

**TABLE 2:** Five-fold cross-validation of the performance of the algorithms in the total dataset.

		**AUC** **(95% CI)**	**Accuracy** **(95% CI)**	**Specificity** **(95% CI)**	**Sensitivity** **(95% CI)**		

Algorithm I		0.995 (0.993, 0.996)	0.973 (0.969, 0.977)	0.981 (0.978, 0.985)	0.939 (0.933, 0.945)		

		**Macro-AUC**	**Accuracy** **(95% CI)**	**Quadratic-weighted kappa** **(95% CI)**			

Algorithm II		0.979 (0.972, 0.985)	0.967 (0.963, 0.971)	0.988 (0.986, 0.990)			

		**Image classification**			**ROI detection and lesion localization**		
	**Classification**	**Accuracy** **(95% CI)**	**Specificity** **(95% CI)**	**Sensitivity** **(95% CI)**	**Recall**	**Precision**	**F1-score**

Algorithm III	CNV	0.970 (0.966, 0.974)	0.970 (0.966, 0.974)	0.973 (0.969, 0.977)	0.916	0.789	0.848
	Fuchs	0.971 (0.967, 0.975)	0.971 (0.967, 0.975)	0.978 (0.975, 0.982)	0.915	0.864	0.889
	LC	0.994 (0.992, 0.995)	0.995 (0.993, 0.996)	0.684 (0.672, 0.695)	0.724	0.656	0.688

	**Accuracy** **(95% CI)**		**Sensitivity** **(95% CI)**	**Specificity** **(95% CI)**		**Precision** **(95% CI)**	

Model-1	0.973 (0.969, 0.977)		0.939 (0.933, 0.945)	0.981 (0.978, 0.985)		0.926 (0.920, 0.933)	
Model-2	0.984 (0.981, 0.987)		0.946 (0.941, 0.952)	0.992 (0.990, 0.995)		0.967 (0.963, 0.972)	

### External Validation and Expert-Machine Comparison

**FIGURE 3:**
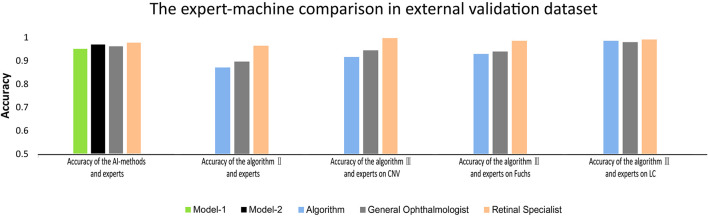
The comparison between deep learning algorithms/AI-models and experts on accuracy in external validation.

Based on the results of better performance in identifying PM, model-2 and algorithms were further evaluated in the external validation dataset (Supplementary Table 1 and Supplementary Figures 2C,D). The performance of model-2 and the three algorithms in the external validation dataset was slightly worse than that in the total dataset. Although there was significant difference in accuracy between the AI-models/deep learning algorithms and experts in terms of identifying PM (*P* = 0.013), distinguishing different MM lesions (*P* < 0.001), and detecting CNV (*P* < 0.001) and Fuchs’ spot (*P* < 0.001), the AI-models/deep learning algorithms achieved an overall comparable performance to that of the experts (Figure 3). For PM identifying, model-2 exhibited even higher accuracy than the general ophthalmologist (96.9 vs. 96.1%). In each task of algorithm II and algorithm III, the difference in accuracy compared to the general ophthalmologist was within 3%. The detailed outcomes of the external validation are shown in Supplementary Table 1.

### Misclassified Image Analysis in the External Validation Dataset

There were 49 images misclassified by algorithm I, including 21 false negatives and 28 false positives. All false negatives were produced in eyes with the other PM complications, such as retinal detachment and retinal vein obstruction. The false positives included 24 tessellated fundus images, 3 proliferative retinopathy images, and 1 exudative retinopathy image. The major error of algorithm II was that 38 Category 0 images were erroneously classified as Category 1 images. Additionally, 23 Category 3 images were identified as Category 4 images. Typical misclassified images are shown in Supplementary Figures 4A–E, and the confusion matrices are given in Supplementary Figures 2C,D.

### Visualization Heatmap Analysis

**FIGURE 4:**
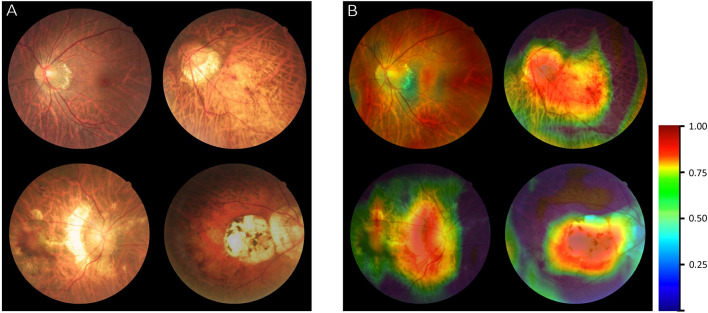
Visualization of algorithm II for classifying the category of myopic maculopathy (MM). **(A)** The original images of different MM (Category 1–Category 4). **(B)** Heatmap generated from deep features overlaid on the original images. The typical MM lesions were observed in the hot regions.

The original images of different MM categories were input into algorithm II as exampled in Figure 4A. After generating a fundus heatmap by the visualization layer, the regions where the algorithm thought most critical for its choice were highlighted in a color scale as shown in Figure 4B. Subsequently, the senior retinal specialist checked the consistency of hot regions highlighted by the algorithm and actual typical MM lesions, including tessellated fundus, diffuse chorioretinal atrophy, patchy chorioretinal atrophy, and macular atrophy. The results by algorithm showed good alignment with the diagnosis by the specialists. Of note, in ophthalmic practice, these lesions are used to diagnose PM.

## Discussion

Based on retinal fundus images, the present work developed a series of deep learning algorithms implementing three tasks: (1) identify PM, (2) classify the category of MM, and (3) localize the “Plus” lesions. After comparing two AI-models comprising the three algorithms, we confirmed that the two-step AI-model (model-2) showed better performance. Although there were still gaps between the AI-models/algorithms and the retinal specialists, metrics of our AI-models/algorithms at this stage were comparable to the general ophthalmologists. Our work was an exploratory and innovative effort to apply deep learning technologies to the diagnosis and management of PM.

Recently, several automatic detection systems for PM have been reported. Tan et al. (2009) introduced the PAMELA system, which could automatically identify PM based on the peripapillary atrophy features. Freire et al. (2020) reported their work of PM diagnosis and detection of retinal structures and some lesions which achieved satisfactory performance both in classification and segmentation tasks. Devda and Eswari (2019) developed a deep learning method with CNN for tasks of Pathologic Myopia Challenge (PALM) based on the dataset provided by International Symposium on Biomedical Imaging (ISBI). Their works showed a better performance when compared to the PAMELA system. However, both of these systems were developed from public databases, such as the Singapore Cohort Study of the Risk factors for Myopia and ISBI. The volumes of the training and test datasets involved in the development process of these systems have been relatively small. Moreover, authoritative criteria for identifying PM were lacking in these studies.

In this work, a large dataset of 37,659 retinal fundus images was used to develop the algorithms. The dataset from real world was able to provide more original disease information and data complexity than public databases. Nevertheless, one major challenge for algorithms is the general applicability to the data and hardware settings outside the development site (Liu et al., 2019). One resolution is to maximize the diversity of data sources so as to prevent parameter overfitting and improve generalizability. For the present work, all the training images were obtained from three hospitals in three different provinces and captured by cameras from three different manufacturers, respectively. Meanwhile, we also constructed an external validation dataset including 1,000 fundus images from the other three additional hospitals to further test our algorithms and AI-models. As expected, the high diversity of the data source lowered the performance of our algorithms somewhat, but the results were still acceptable, with the accuracy of 96.9%, sensitivity of 98.8%, and specificity of 94.6% for model-2 especially, justifying the validity of our algorithms.

This study applied a systemic classification standard of META-PM, which was widely applied in clinical trials and epidemiologic studies. Unlike the criteria used in other studies, META-PM classification is not only simpler but also has more clinical implications. The category of MM can reflect the severity of PM to a large extent, as the morphological and functional characteristics of highly myopic eyes were found to be positively correlated with MM category (from Category 0 to 3) (Zhao et al., 2020). The “Plus” lesions can be concurrent with any MM category and have a significant impact on vision (Wong et al., 2015). Therefore, our algorithms and AI-models can assist the clinicians with valuable and practical fundus information of PM.

There was a similar study that applied the META-PM classification. Du et al. (2021) reported a META-PM categorizing system (META-PM CS) integrating four DL algorithms and a special processing layer. This system could recognize the fundus images of Category 2 to 4 MM and CNV and detect PM defined as having MM equal to or more serious than diffuse atrophy (category 2). Compared with their system, our deep learning algorithms are more powerful and can automatically classify the category of MM and localize the “Plus” lesions based on retinal fundus images. Our AI-model could obtain the output of the algorithms mentioned above and use the logical analysis module to analyze the results of algorithms to determine whether the image was of PM. The core of the logical analysis module was the more precise PM definition (equal to or more serious than diffuse atrophy (category 2) or with at least one of the “plus” lesions) based on the META-PM classification.

In addition to the large training dataset and systemic META-PM standard, the other advantage of our work is the CNN architecture selected for development of algorithms and AI-models. The ResNet18 was used as the basic architecture for all the classification algorithms, and the Faster R-CNN+FPN was used for the localization algorithm in our work. ResNet was proposed in 2015 after three classical CNN networks, namely, AlexNet, GoogLeNet, and VGG were established and had won the top prize in the ImageNet competition classification task. ResNet is arguably the most pioneering work in computer vision and deep learning in the past few years, as it can effectively solve the problem of accuracy saturation and decline while the network depth increases by introducing a shortcut mechanism (He et al., 2016; Zhu et al., 2019). Faster R-CNN is one of the most advanced object detection networks, which can integrate the feature and proposal extractions as well as the classification and bounding box regression (Ren et al., 2017; Ding et al., 2020). FPN, a densely connected feature pyramid network, can build high-level semantic feature maps at all scales for object detection (Tayara and Chong, 2018; Pan et al., 2019). With the same backbone network, the FPN-based Faster R-CNN system is thought to be superior to all existing single-model entries (Pan et al., 2019). Therefore, our algorithms and AI-models were based on the advanced architectures and should be precise and efficient for the PM detection.

The distribution of misclassifications by our algorithms was also analyzed in the external validation dataset. The misclassified images generated by algorithm I were mainly due to the misjudgment of certain diseases that appeared less frequently in training. Meanwhile, algorithm II made some errors in distinguishing Category 3 and Category 4 MM. To minimize the errors, increasing the images of specific diseases into the training dataset and applying visual attention mechanisms to the CNN architecture will always be an effective approach (Pesce et al., 2019). Moreover, the visualization results demonstrated that the typical MM lesions of each category appeared in the regions where the algorithm made a positive contribution to the classification results, so that our algorithm is justified to be convincing from the clinical point of view. These results also indicated the directions of optimization and updating for the algorithms in our future’s work.

This study still has limitations. First, our algorithm had the ability to automatically detect and localize “Plus” lesions, but the performance metrics were slightly lower if compared with that in the mission of MM classification, especially for the LC detection. LCs vary greatly in shape, size, color, and location. Combining with infrared reflectance or indocyanine green angiography image is certainly the more effective method to detect LCs than using the fundus images alone. However, the fundus images are relatively easy and economical for clinical practice in most medical institutes. At this stage, the “plus” lesions appeared in less than 15% of images containing MM of all severity in the total dataset. With the continuously accumulated data by our work, the performance of the algorithm will be further improved. Second, the presence of posterior staphyloma is also defined as PM according to the META-PM classification (Ohno-Matsui et al., 2016), but it is difficult to diagnose posterior staphyloma accurately from fundus images; MRI or OCT images are needed. Our algorithm does not yet have the capability to detect or localize posterior staphyloma. A multimodal imaging AI diagnostic platform involving fundus images, OCT, optical coherence tomography angiography (OCTA), fluorescein angiography, and MRI data is our ongoing effort to establish a more powerful automatic system to identify PM lesions (Ruiz-Medrano et al., 2019).

In conclusion, this study developed a series of deep learning algorithms and AI-models that have the ability to automatically identify PM, classify the category of MM, and localize the “Plus” lesions based on retinal fundus images. They have achieved performance comparable to that of experts. Due to such promising performance at this stage, we initiated the task of engineering relevant algorithms and hope that our research can make more contributions to clinical and healthcare screening work for myopia patients.

## Data Availability Statement

The raw data supporting the conclusions of this article will be made available by the authors, without undue reservation.

## Ethics Statement

Written informed consent was not obtained from the individual(s) for the publication of any potentially identifiable images or data included in this article. As the study was a retrospective review and analysis of fully anonymized retinal fundus images, the medical ethics committee declared it exempt from informed consent.

## Author Contributions

LL and WH: design of the study. MYu, WY, JH, EZ, QL, XT, PR, QH, MZ, and GK: acquisition, analysis, or interpretation of the data. LL, PR, and XT: drafting of the manuscript. LL and LXL: development of the algorithms and models. LL, MY, WY, and QH: statistical analysis. LL and WH: obtaining the fund. WH: supervising the process. All authors: revision and approval of the manuscript.

## Conflict of Interest

The authors declare that the research was conducted in the absence of any commercial or financial relationships that could be construed as a potential conflict of interest.

## Publisher’s Note

All claims expressed in this article are solely those of the authors and do not necessarily represent those of their affiliated organizations, or those of the publisher, the editors and the reviewers. Any product that may be evaluated in this article, or claim that may be made by its manufacturer, is not guaranteed or endorsed by the publisher.
