# Supplementary material for: AI-Model for Identifying Pathologic Myopia Based on Deep Learning Algorithms of Myopic Maculopathy Classification and “Plus” Lesion Detection in Fundus Images
Source: Front Cell Dev Biol. 2021 Oct 15;9:719262. doi: 10.3389/fcell.2021.719262 (PMC8554089; doi:10.3389/fcell.2021.719262)
Supplement: Supplementary file 1 [file Table_1.DOCX]

**Supplementary Table 1. Comparison of the Performance of the Algorithms and Experts in External Validation Dataset**

|  |  | **AUC**  **(95% CI)** | **Accuracy**  **(95% CI)** | **Specificity**  **(95% CI)** | **Sensitivity**  **(95% CI)** |  |  |
| --- | --- | --- | --- | --- | --- | --- | --- |
| Algorithm I |  | 0.989  (0.986, 0.991) | 0.951  (0.937, 0.964) | 0.935  (0.920, 0.950) | 0.963  (0.951, 0.974) |  |  |
| General ophthalmologist |  | NA | 0.961  (0.949, 0.973) | 0.973  (0.963, 0.983) | 0.944  (0.930, 0.958) |  |  |
| Retinal specialist |  | NA | 0.977  (0.967, 0.986) | 0.984  (0.976, 0.991) | 0.967  (0.956, 0.978) |  |  |
|  |  | **Macro-AUC** | **Accuracy**  **(95% CI)** | **Quadratic-weighted kappa**  **(95% CI)** | |  |  |
| Algorithm II |  | 0.914  (0.900, 0.927) | 0.870  (0.849, 0.890) | 0.978  (0.973, 0.983) | |  |  |
| General ophthalmologist |  | NA | 0.896  (0.877, 0.914) | 0.990  (0.987, 0.993) | |  |  |
| Retinal specialist |  | NA | 0.963  (0.951, 0.974) | 0.995  (0.993, 0.998) | |  |  |
|  |  | **Image classification** | | | **ROI detection and lesion localization** | | |
|  | Classification | Accuracy  (95% CI) | Specificity  (95% CI) | Sensitivity  (95% CI) | Recall | Precision | F1-score |
| Algorithm III | CNV | 0.915  (0.897, 0.932) | 0.915  (0.898, 0.932) | 0.910  (0.892, 0.928) | 0.897 | 0.770 | 0.829 |
|  | Fuchs | 0.928  (0.912, 0.944) | 0.914  (0.897, 0.931) | 0.980  (0.972, 0.989) | 0.928 | 0.829 | 0.876 |
|  | LC | 0.984  (0.976, 0.991) | 0.985  (0.977, 0.992) | 0.888  (0.869, 0.908) | 0.833 | 0.645 | 0.727 |
|  |  | **Image classification** | | | **ROI detection and lesion localization** | | |
| General ophthalmologist | CNV | 0.943  (0.928, 0.957) | 0.943  (0.928, 0.957) | 0.940  (0.925, 0.955) | 0.959 | 0.930 | 0.944 |
|  | Fuchs | 0.938  (0.923, 0.952) | 0.928  (0.912, 0.944) | 0.975  (0.966, 0.985) | 0.967 | 0.929 | 0.948 |
|  | LC | 0.978  (0.968, 0.987) | 0.979  (0.971, 0.988) | 0.777  (0.752, 0.803) | 0.708 | 0.773 | 0.739 |
| Retinal specialist | CNV | 0.995  (0.990, 0.999) | 0.996  (0.993, 1.000) | 0.970  (0.959, 0.980) | 0.979 | 0.969 | 0.974 |
|  | Fuchs | 0.984  (0.976, 0.991) | 0.983  (0.975, 0.991) | 0.985  (0.977, 0.992) | 0.980 | 0.948 | 0.964 |
|  | LC | 0.989  (0.982, 0.995) | 0.988  (0.982, 0.995) | 1.000  (1.000, 1.000) | 0.792 | 0.826 | 0.809 |
|  |  | **Accuracy**  **(95% CI)** | **Specificity**  **(95% CI)** | **Sensitivity**  **(95% CI)** | **Precision**  **(95% CI)** |  |  |
| Model-1 |  | 0.951  (0.937, 0.964) | 0.935  (0.920, 0.950) | 0.963  (0.951, 0.974) | 0.951  (0.937, 0.964) |  |  |
| Model-2 |  | 0.969  (0.958, 0.979) | 0.945  (0.931, 0.959) | 0.987  (0.980, 0.994) | 0.958  (0.946, 0.970) |  |  |
| General ophthalmologist |  | 0.961  (0.949, 0.973) | 0.973  (0.963, 0.983) | 0.944  (0.930, 0.958) | 0.967  (0.956, 0.978) |  |  |
| Retinal specialist |  | 0.977  (0.967, 0.986) | 0.984  (0.976, 0.991) | 0.967  (0.956, 0.978) | 0.964  (0.953, 0.976) |  |  |
