# Supplementary figures and images for: AI-Model for Identifying Pathologic Myopia Based on Deep Learning Algorithms of Myopic Maculopathy Classification and “Plus” Lesion Detection in Fundus Images
Source: Front Cell Dev Biol. 2021 Oct 15;9:719262. doi: 10.3389/fcell.2021.719262 (PMC8554089; doi:10.3389/fcell.2021.719262)

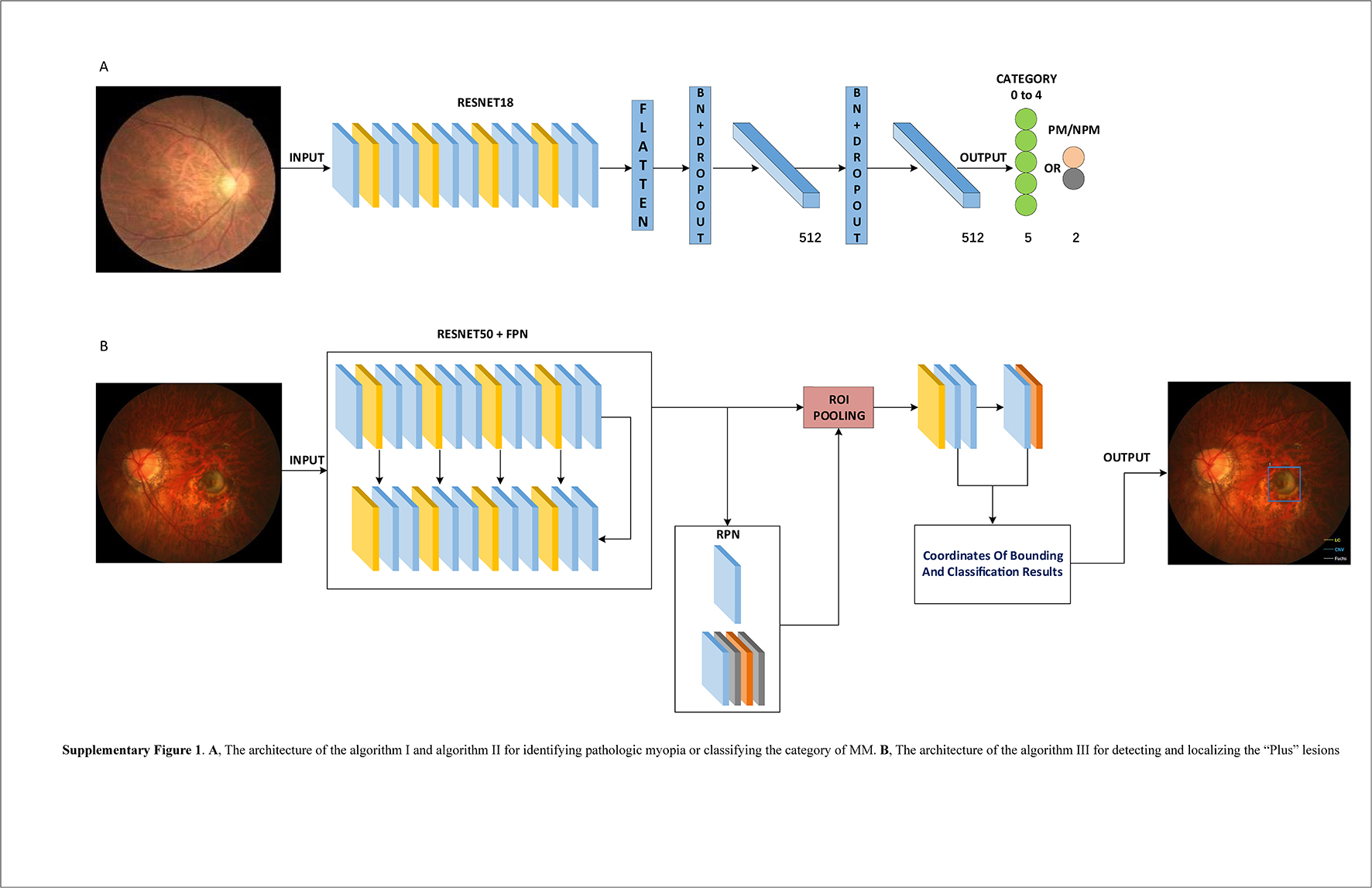

Supplement: Supplementary file 2 [file Image_1.JPEG]

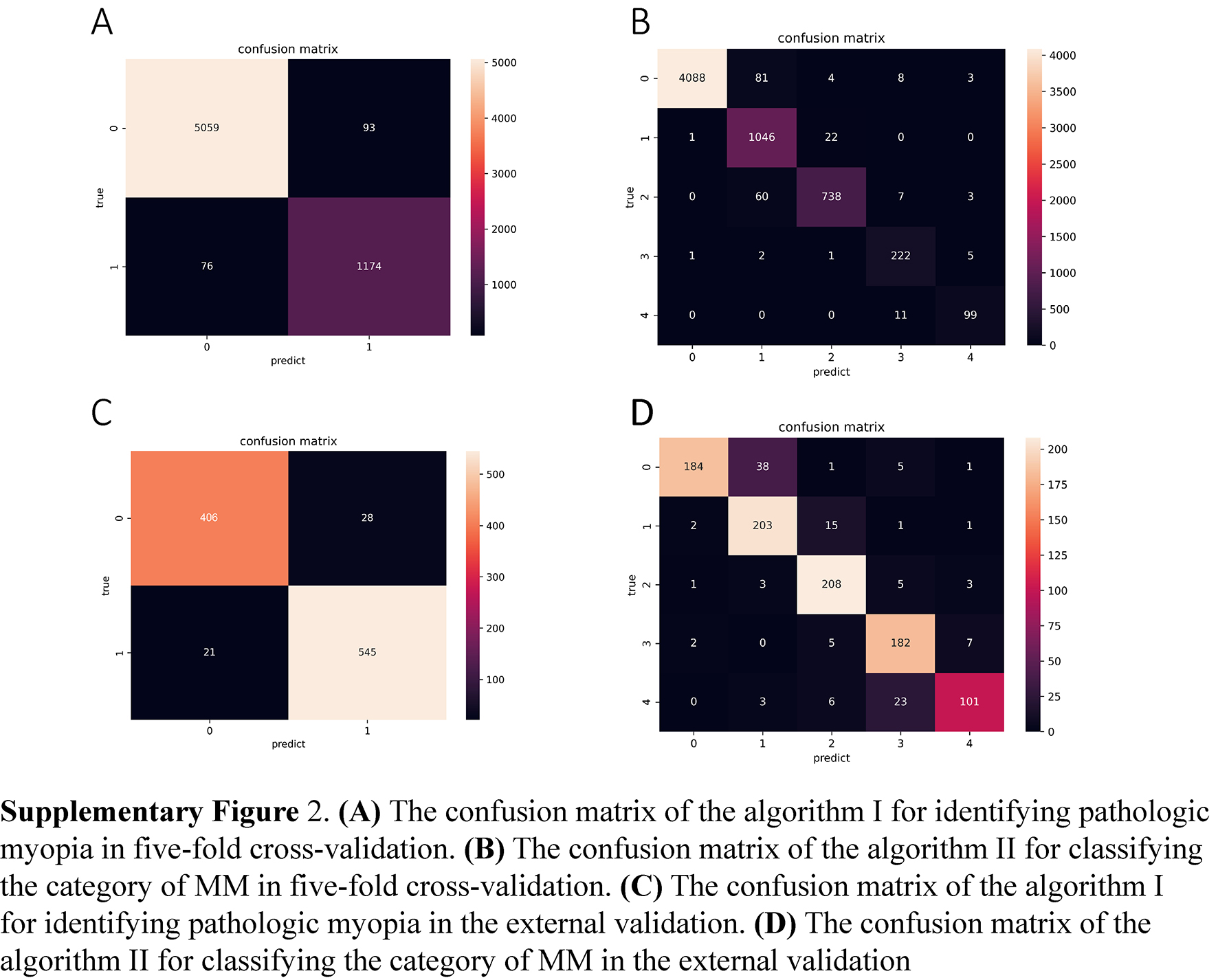

Supplement: Supplementary file 3 [file Image_2.JPEG]

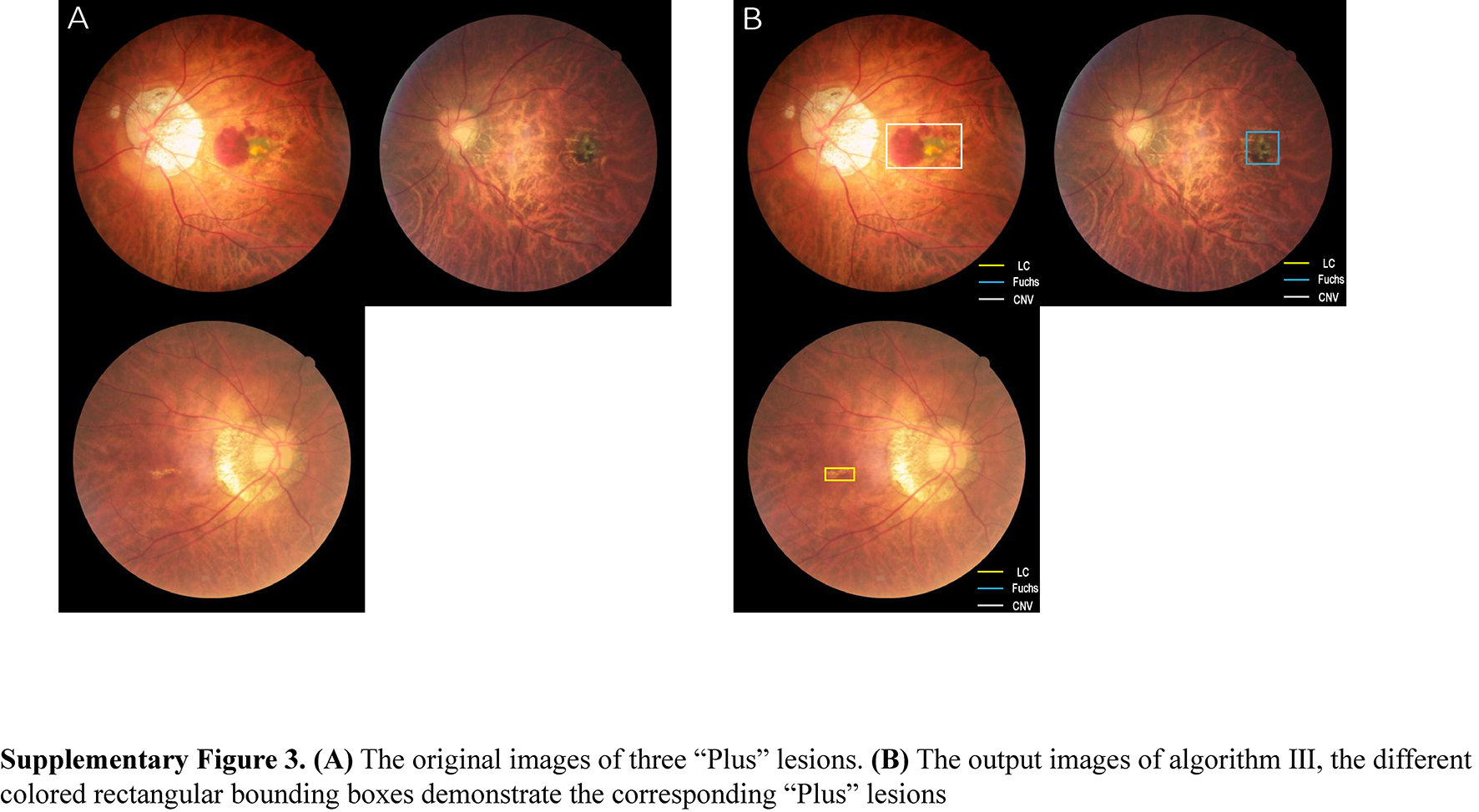

Supplement: Supplementary file 4 [file Image_3.JPEG]

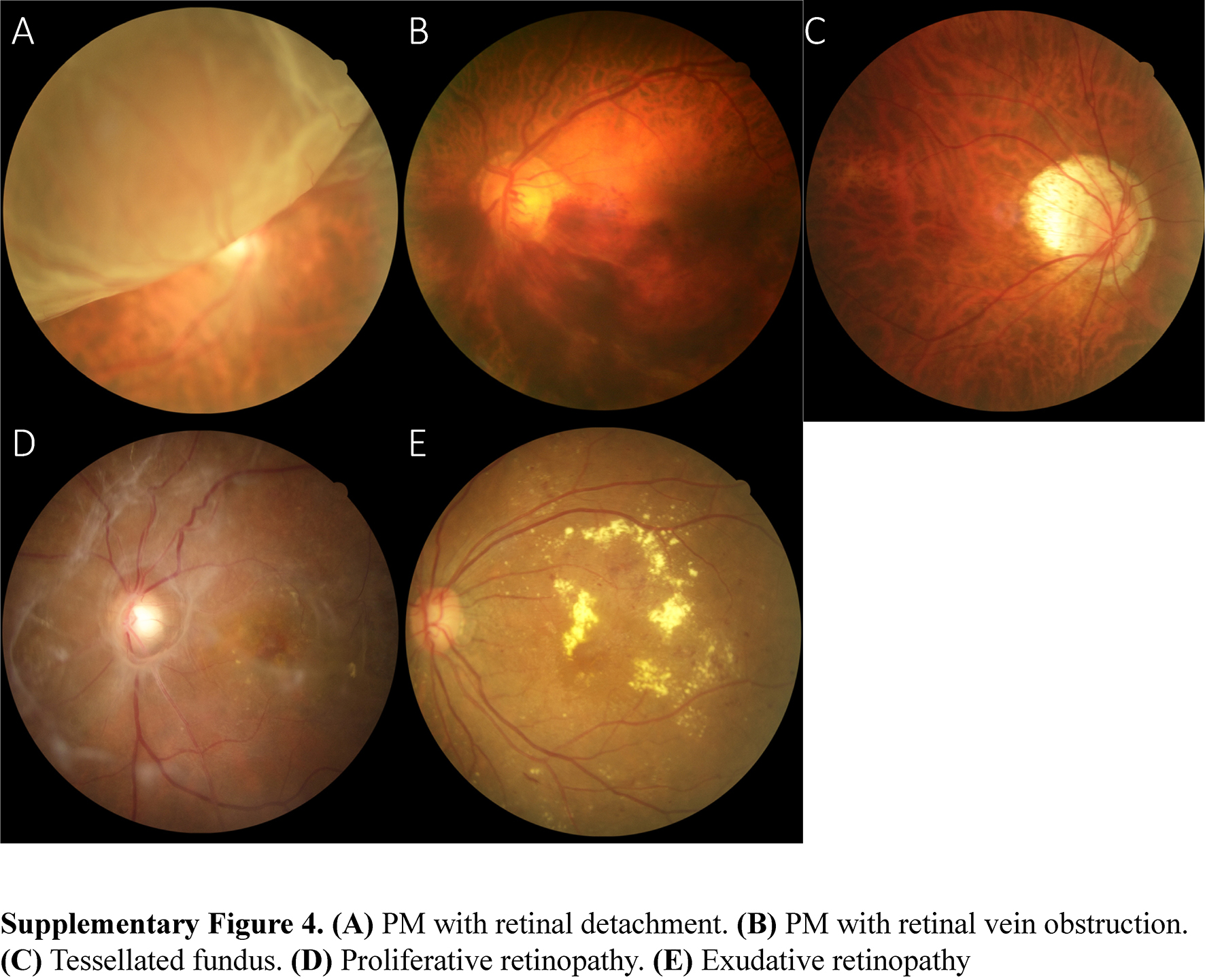

Supplement: Supplementary file 5 [file Image_4.JPEG]
